# A Perspective for Developing Polymer-Based Electromagnetic Interference Shielding Composites

**DOI:** 10.1007/s40820-022-00843-3

**Published:** 2022-04-01

**Authors:** Yali Zhang, Junwei Gu

**Affiliations:** Shaanxi Key Laboratory of Macromolecular Science and Technology, School of Chemistry and Chemical Engineering, Northwestern Polytechnical University, Xi’an, 710072 Shaanxi People’s Republic of China

**Keywords:** Polymer composites, Electromagnetic interference shielding, Conductive network, Lightweight

## Abstract

The rapid development of aerospace weapons and equipment, wireless base stations and 5G communication technologies has put forward newer and higher requirements for the comprehensive performances of polymer-based electromagnetic interference (EMI) shielding composites. However, most of currently prepared polymer-based EMI shielding composites are still difficult to combine high performance and multi-functionality. In response to this, based on the research works of relevant researchers as well as our research group, three possible directions to break through the above bottlenecks are proposed, including construction of efficient conductive networks, optimization of multi-interfaces for lightweight and multifunction compatibility design. The future development trends in three directions are prospected, and it is hoped to provide certain theoretical basis and technical guidance for the preparation, research and development of polymer-based EMI shielding composites.

## Highlights

- Bottlenecks for developing polymer based electromagnetic interference (EMI) shielding composites are proposed and inner reasons are discussed
- Possible directions to break through bottlenecks are raised and recent advances in such directions are introduced.
- Development trends in the future are foreseen to provide theoretical basis and technical guidance for development of polymer based EMI shielding composites.

## Graphical abstract

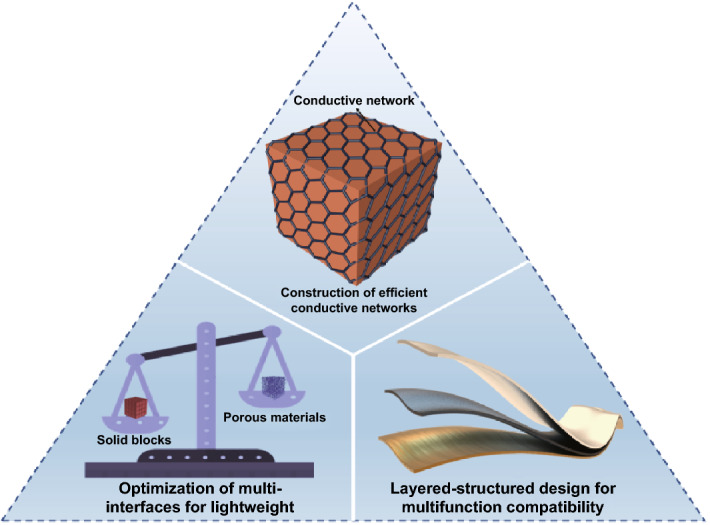

## Introduction

The rapid development of aerospace weapons and equipment, wireless base stations and 5G communication technologies has led to the upgrading of electronic equipments and components toward high frequency, high power, high density and high integration, which inevitably brings problems such as electromagnetic radiation and electromagnetic interference (EMI), seriously affecting the normal operation of electronic equipments and components, safe transmission of information, as well as human health [1–3]. The EMI shielding materials can effectively protect electronic equipments and their environment, prevent electromagnetic information leakage, cut off propagation paths of electromagnetic waves and suppress radiation and interference of electromagnetic waves. It is one of the most important technical means to solve the problem of electromagnetic radiation and electromagnetic pollution [4, 5]. With the development of new generation of electronic equipment in the direction of intelligence, portability and wearability, higher requirements are also placed on the lightweight, flexibility, thermal conductivity and mechanical properties of EMI shielding materials. Therefore, the research and development of novel high-performance and multi-functional EMI shielding composites with excellent comprehensive properties have urgent theoretical significance and practical application value for the design and expansion of materials in fields related to aerospace, wearable electronic devices and artificial intelligence [6–8].

Polymer-based EMI shielding composites are gradually replacing traditional metal-based EMI shielding materials because of their lightweight, high specific strength, corrosion resistance, easy processing, and excellent EMI shielding performances. The shielding effects of polymer-based EMI shielding composites on electromagnetic waves are mainly realized by introducing electrically/magnetically conductive fillers into the polymer matrix to form the electrically/magnetically conductive network [9, 10]. At present, most of the polymer-based EMI shielding composites are prepared by solution blending, melt blending, *in situ* polymerization, etc*.* In these methods, the polymer matrix and functional fillers are directly mixed, and the functional fillers in the composites are randomly distributed in the entire polymer matrix. It is not conducive to the efficient construction of the electrically/magnetically conductive network, resulting in very limited EMI shielding ability [11]. To achieve the desired EMI shielding effectiveness (EMI SE), large amount of electrically/magnetically conductive fillers are usually required [12]. However, high filling of functional fillers will seriously affect the processability and mechanical properties of polymer-based EMI shielding composites. Researchers have improved the compatibility and dispersion of electrically/magnetically conductive fillers in polymer matrix by functionalization via covalent bonds or non-covalent bonds, but the improvement in electrical conductivity (*σ*) and EMI SE is still relatively limited [13, 14]. Especially in aerospace, wearable electronic devices, artificial intelligence and other fields, not only excellent EMI shielding performance and mechanical properties are required, lightweight and multi-functionality are also the key technical indicators [15, 16]. However, most of the currently prepared polymer-based EMI shielding composites are difficult to meet all these requirements.

Our research group has been focusing our studies on the structural design and preparation of polymer-based EMI shielding composites. Based on multi-scale network structure design, functional filler–polymer matrix interface regulation and structure/function integration, a series of polymer-based EMI shielding composites have been prepared. The internal relationship between their microstructures and macroscopic properties is explored. This paper aims to put forward the research ideas and technical routes that can be taken in the future in view of the bottleneck problems in the field of polymer-based EMI shielding composites, hoping to provide certain theoretical basis and technical guidance for the preparation, research and development of polymer-based EMI shielding composites.

## Possible Directions for Developing Polymer-Based EMI Shielding Composites

### Construction of Efficient Conductive Networks

Polymer-based EMI shielding composites prepared by simple blending method are usually difficult to achieve excellent EMI shielding performance at low amount of conductive fillers [17, 18]. Gathering conductive fillers at the interfaces among the polymer particles through electrostatic interaction, or backfilling the polymer matrix into the constructed three-dimensional skeleton of conductive fillers, so as to achieve efficient overlap between the conductive fillers, forming more efficient and complete conductive networks, are the interesting ideas to realize excellent electrical conductivities and EMI shielding performances of polymer-based EMI shielding composites with relatively low amount of conductive fillers [19].

**Fig. 1:**
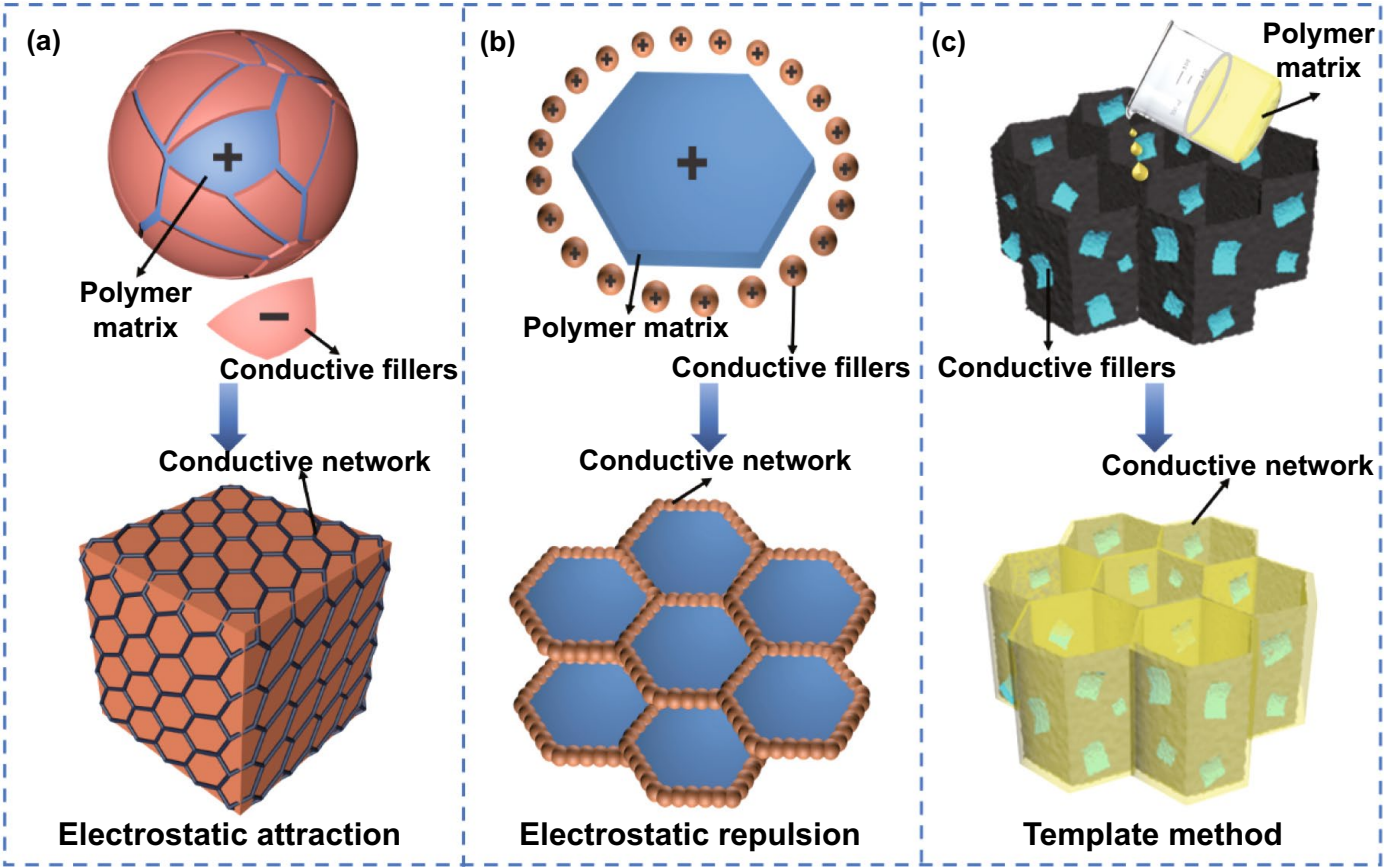
Schematic illustration of constructing efficient conductive networks by **a** electrostatic attraction, **b** electrostatic repulsion and **c** template method

Electrostatic interaction forces can control the distribution state of nano-sized conductive fillers among polymer particles, enabling precise control of efficient three-dimensional conductive networks. By electrostatic attraction, negatively (positively) charged nano-sized conductive fillers selectively distribute on the surface of positively (negatively) charged polymer particles, rather than inside the entire polymer matrix, and precisely self-assemble to form efficient conductive network, Fig. 1a. By electrostatic repulsion, nano-sized conductive fillers with the same charges are squeezed out at interfaces between the polymer particles. By increasing the content of polymer particles or applying external force, the nano-sized conductive fillers are tightly packed at the interfaces, thereby forming the uniform and complete highly efficient conductive network, Fig. 1b. Sun et al*.* [20] used two-dimensional layered transition metal carbide (MXene) as highly conductive fillers, uniformly attached negatively charged MXene nanosheets to the surface of positively charged polystyrene (PS) microspheres through electrostatic interaction, and prepared MXene@PS EMI shielding composites with continuous and efficient conductive network by molding. Benefiting from the continuous, regular and efficient conductive network inside MXene@PS EMI shielding composites, when the volume fraction of MXene was only 1.9 vol%, the *σ* was 1081 S m^−1^, much higher than that of MXene/PS EMI shielding composites prepared by solution blending method (2.2 × 10^–7^ S m^−1^), and its EMI SE was as high as 61 dB. Luo et al*.* [21] used electrostatic repulsion to self-assemble negatively charged natural rubber (NR) and negatively charged MXene with vacuum-assisted filtration to obtain MXene/NR EMI shielding composite films. The MXene nanosheets were selectively distributed on the interfaces between NR particles via electrostatic repulsion, forming the interconnected honeycomb-like efficient conductive network. When the volume fraction of MXene was 6.71 vol%, its *σ* and EMI SE were as high as 1400 S m^−1^ and 53 dB, respectively.

In addition, microstructure design of conductive fillers by template method, inducing the serial distribution of fillers and assembly into three-dimensional skeleton of conductive fillers and then backfilling polymer matrix into skeletons by vacuum-assisted impregnation, is also an effective way to construct efficient conductive networks, Fig. 1c. Three-dimensional skeleton structure can help the conductive fillers to be distributed in the polymer matrix, realize the efficient overlapping of the conductive networks and then efficiently improve the conductive loss of electromagnetic waves. And at the same time, the interfaces between conductive fillers and polymer matrix can be used to enhance the regular reflection and reabsorption of the internal electromagnetic waves, and the efficient construction of the internal conductive networks and the rapid improvement in EMI SE of the polymer-based EMI shielding composites under low amount of conductive fillers can be achieved [22, 23].

Zhao et al*.* [24] prepared three-dimensional highly conductive MXene/reduced graphene oxide (rGO) aerogel frameworks with directionally porous structure by means of graphene oxide (GO)-assisted hydrothermal assembly and directional freeze-drying. Then, MXene/rGO/epoxy EMI shielding composites were prepared by backfilling epoxy resin by vacuum-assisted impregnation. When the volume fraction of MXene was 0.74 vol%, the *σ* and EMI SE of MXene/rGO/epoxy EMI shielding composites reached 695.9 S m^−1^ and 56 dB, respectively. Shen et al*.* [25] prepared the carbon skeleton with continuous structure by delignification and carbonization of natural wood and prepared wood-derived carbon/epoxy composites after backfilling epoxy resin. Results showed that when the carbon content was 7.0 vol%, the *σ* and EMI SE of the epoxy/wood-derived carbon composite were 12.5 S m^−1^ and 27.8 dB, respectively. In our previous research work, Gu et al*.* [26] prepared reduced graphene oxide (rGO)-MXene with honeycomb structure (rGMH) by combination of template (honeycomb made of aluminum oxide) induction and electrostatic self-assembly and backfilled epoxy resin to obtain rGMH/epoxy EMI shielding composites. Honeycomb structure has special hexagonal structure, i.e., a structure of cells with six sides, like the hive made by bees for holding their honey. On the one hand, its regular structure made the conductive fillers arrange in the orderly manner and overlap efficiently, so as to realize the construction of efficient conductive pathways. On the other hand, its hexagonal structure enabled electromagnetic waves to undergo multiple reflection, scattering and absorption processes, prolonging the transmission distance of electromagnetic waves in the composite materials and improving the EMI shielding performances. When the amounts of rGO and MXene were 1.2 wt% and 3.3 wt%, respectively, the *σ* and EMI SE of rGMH/epoxy EMI shielding composites were as high as 387.1 S m^−1^ and 55 dB, respectively, 2978 and 5 × those of the blended rGO-MXene/epoxy composites with the same amounts of fillers (*σ* and EMI SE were 0.13 S m^−1^ and 11 dB, respectively). In addition, Gu et al*.* [27] prepared silver platelets (AgPs)/rGO foam with spherical hollow structure by sol–gel templating method and backfilled epoxy resin to prepare AgPs/rGO/epoxy composites. Results showed that, benefiting from the construction of three-dimensional conductive networks, when the thickness was 3 mm and the volume fractions of rGO and AgPs were 0.44 vol% and 0.94 vol%, respectively, the EMI SE of AgPs/rGO/epoxy composites reached 58 dB at *X*-band.

The construction of high-efficiency conductive networks can improve the effective overlap between conductive fillers and further reduce the percolation threshold of composites. On the other hand, the fillers/polymer interfaces can be used more efficiently to enhance the reflection and reabsorption of electromagnetic waves between ordered structures and effectively improve the loss ability of electromagnetic waves. However, the polymer-based EMI shielding composites prepared based on the three-dimensional efficient conductive network only have weak electrostatic interaction or hydrogen bond interaction between the polymer matrix and the conductive fillers, resulting in poor mechanical properties and difficult to achieve practical application requirements. In the future research work, researchers can try to enhance the interaction between the two by means of chemical bonding through the optimal selection and combination design of polymer matrix and functional fillers, and improve the mechanical properties of composites. In addition, the construction of the three-dimensional conductive network endows the polymer-based EMI shielding composites with excellent electrical conductivity, and its EMI shielding mechanism is mostly based on reflection, which is easy to cause new EMI problems. The introduction of magnetic fillers and the construction of absorption-based microstructures to achieve absorption-based EMI shielding are also issues for researchers to consider.

### Optimization of Multi-interfaces for Lightweight

The densities of polymer-based EMI shielding composites are significantly lower than those of traditional metal EMI shielding materials, but it is still difficult to meet the low density expectations required in fields such as aerospace [28, 29]. The density of composites can be effectively reduced by optimization of multi-interfaces, which is also a hopeful idea for the preparation of lightweight and high-performance polymer-based EMI shielding composites [30].

**Fig. 2:**
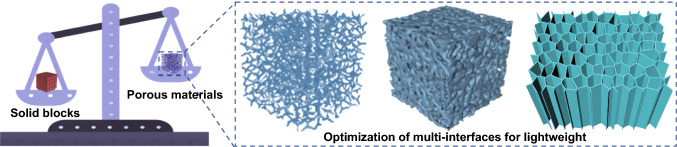
Schematic diagram of optimization of multi-interfaces for lightweight

The optimization of multi-interfaces based on the porous structure can not only reduce the density of the composites, but also realize the multiple reflection and scattering loss of the electromagnetic waves, prolong the transmission paths of the electromagnetic waves and further improve the EMI shielding performances, Fig. 2. Zeng et al*.* [31] blended multi-walled carbon nanotubes (MWCNT) and waterborne polyurethane (WPU) solution to prepare lightweight MWCNT/WPU aerogels with anisotropic pore structure by freeze-drying technology. When the mass fraction of MWCNT was 76.2 wt%, the EMI SE of the MWCNT/WPU aerogel reached 50 dB and the density was only 126 mg cm^−3^. Lu et al*.* [32] prepared ultralight GO/lignin aerogels with oriented microporous structure by directional freeze-drying technology and then thermally reduced them to obtain rGO/lignin-derived carbon (rGO/LDC) EMI shielding composite aerogels. Benefiting from the establishment of the ordered porous structure, the rGO/LDC composite aerogel achieved the EMI SE of 49 dB at ultra-low density of 8 mg cm^−3^. In our previous research work, Gu et al*.* [33] used natural wood as template to obtain wood-derived porous carbon (WPC) skeletons with highly ordered internal pore structure through high-temperature carbonization and then prepared lightweight MXene aerogel/WPC EMI shielding composites by vacuum-assisted impregnation and freeze-drying. When the carbonization temperature was 1500 °C, the MXene aerogel/WPC EMI shielding composites achieved the EMI SE as high as 71 dB at low density of 0.197 g cm^−3^. Therefore, the construction of porous structures not only contributes to the realization of low-density and lightweight of composites, but also contributes to the effective multiple reflection and absorption of electromagnetic waves.

Aerogels prepared by optimization of multi-interfaces generally suffer from poor structural stability and relatively weak controllability on distribution of pore sizes, resulting in relatively poor mechanical properties and shielding stability of polymer-based EMI shielding composites. It is necessary to explore new preparation methods and routes, such as photolithography, microfluidics and additive manufacturing, to optimize and control the pore structure, shape and pore size distribution, and to realize the internal microstructure of polymer-based EMI shielding composites. In addition, how to introduce some reinforcing phases into the composites system to improve its structural stability without affecting EMI shielding performance is also a question worth considering.

### Multifunction Compatibility Design

At present, most research works mainly focus on the improvement in EMI SE of polymer-based EMI shielding composites. The rapid development of modern electronic technology has put forward newer and higher requirements for polymer-based EMI shielding composites. In addition to ensuring its excellent EMI shielding performance, it also needs to have good flexibility, thermal conductivity, self-heating performance and mechanical properties [34, 35]. By introducing multifunctional fillers and constructing the layered structure, it is feasible to realize the multifunctional compatibility design of polymer-based EMI shielding composites [36, 37].

**Fig. 3:**
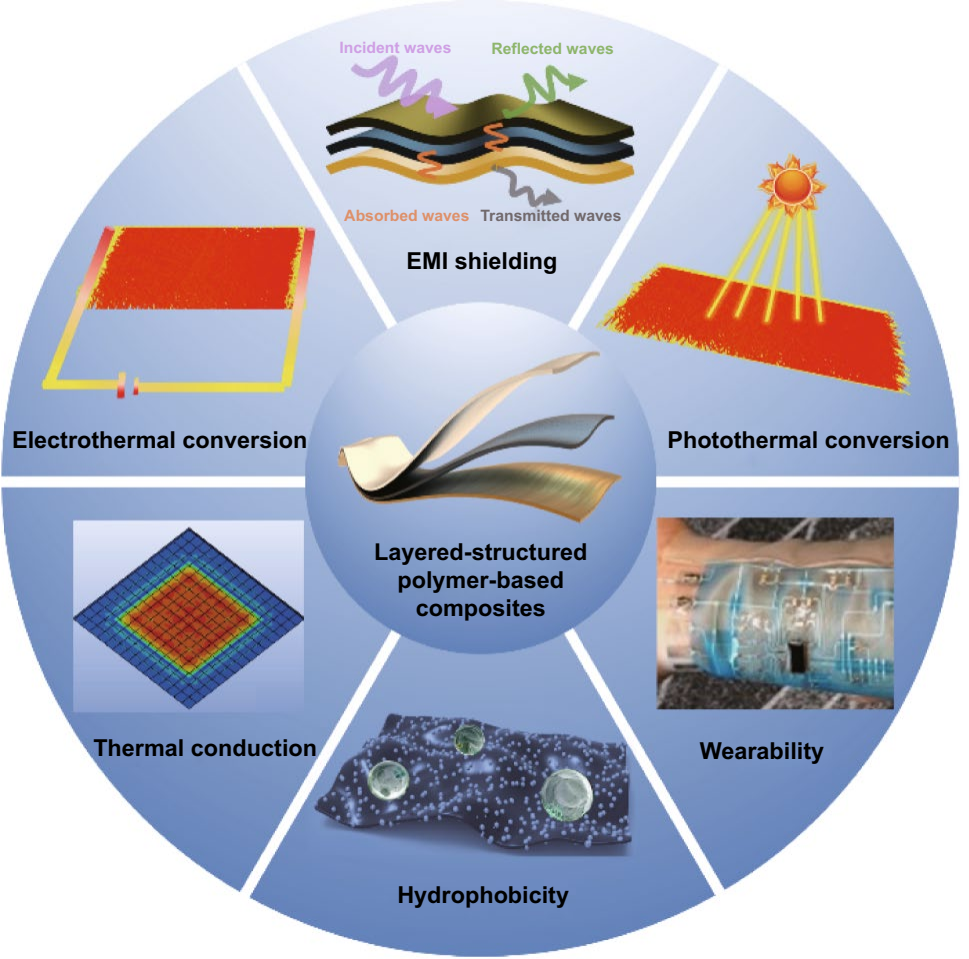
Schematic diagram of multifunction compatibility design for layered-structured polymer-based EMI shielding composites

The layered structure design can concentrate functional fillers in one or more layers, effectively increase the relative concentration of functional fillers and realize the optimal design and combination of various function of different functional fillers, Fig. 3. Zhou et al*.* [38] prepared multifunctional multilayered MXene/CNF/BC composite films by repeatedly spraying MXene/CNF on bacterial cellulose (BC), which was encapsulated with silicone to impart excellent hydrophobicity and solvent resistance. When sprayed with 200 layers of MXene/CNF, the EMI SE of the composite films was as high as 60 dB. In addition, due to the excellent electrical conductivity, extremely strong light absorption and localized surface plasmon resonance (LSPR) effect of MXene, the corresponding composite films had excellent low-voltage driven Joule heating and good photosensitive heating. The dense layered structure and strong interfacial interaction also endowed the composite films with high mechanical strength (> 250 MPa) and good toughness (> 20 MJ cm^−3^). In our previous research work, Gu et al*.* [39] used the facile and efficient two-step vacuum-assisted filtration, hot pressing method to prepare flexible and strong double-layered MXene/silver nanowires-aramid nanofibers (MXene/AgNW-ANF) EMI shielding composite films. The MXene/AgNW-ANF composite film had excellent EMI shielding performance (*σ* and EMI SE up to 3725.6 S cm^−1^ and 80 dB, respectively), excellent Joule heating performance (heating temperature of the composite film up to 110 °C under the low applied voltage of 2.5 V) and mechanical properties (tensile strength up to 235.9 MPa). In addition, Gu et al*.* [40] prepared multifunctional layered AgNW/leather composites with the functions of visual Joule heating, EMI shielding and piezoresistive sensing by simple and efficient vacuum-assisted filtration process using natural leather as the substrate. At the low AgNW area density of 2.5 g m^−2^, the Joule heating temperature of AgNW/leather composites reached 108 °C under the low applied voltage of 2.0 V, the EMI SE reached 55 dB, the square resistance was only 0.8 *Ω* sq^−1^, and it exhibited highly sensitive piezoresistive sensing ability (response time less than 50 ms) in human motion recognition. The AgNW/leather composites also possessed excellent heat resistance, tensile strength, hydrophobicity and working stability.

During multifunctional compatibility design of polymer-based EMI shielding composites, the properties and functions may restrict each other. For example, the optoelectronic system of military weapons and equipment has high demand for the light transmittance of polymer-based EMI shielding composites, but the light transmittance and EMI shielding performance are mutually restricted. How to realize the optimized preparation of high-performance transparent polymer-based EMI shielding composites based on in-depth research on materials and structures is one of the key issues to be solved urgently. In addition, the flexible multifunctional polymer-based EMI shielding composites will be bent, worn or even broken during long-term service, resulting in a great decrease in the electrical conductivities and EMI shielding performances. If researchers introduce the self-healing function into the design and preparation process of flexible multifunctional polymer-based EMI shielding composites, the durability and stability of composites will be greatly improved.

## Summary

In conclusion, although some progresses have been made in the preparation of polymer-based EMI shielding composites, their performance and multifunction compatibility are still far from expectation. In response to this, based on the research works of relevant researchers as well as our research group, three possible directions to break through the above bottlenecks are proposed, including construction of efficient conductive networks, optimization of multi-interfaces for lightweight and multifunction compatibility design. And the research ideas and technical routes that can be taken in the future are put forward, hoping to provide certain theoretical basis and technical guidance for the preparation, research and development of polymer-based EMI shielding composites. It is believed that after breaking through the current bottleneck technical problems, polymer-based EMI shielding composites will play an irreplaceable role in various fields such as aerospace, wearable electronic devices and artificial intelligence.
